# Doing palliative care research on hematologic cancer patients: A realist synthesis of literature and experts’ opinion on what works, for whom and in what circumstances

**DOI:** 10.3389/fonc.2023.991791

**Published:** 2023-03-27

**Authors:** Silvia Tanzi, Gianfranco Martucci

**Affiliations:** ^1^ Palliative Care Unit, Azienda USL-IRCCS Reggio Emilia, Reggio Emilia, Italy; ^2^ Palliative Care Local Program, Local Health Unit of Modena, Modena, Italy

**Keywords:** realist sinthesys, hematologic palliative care, research in hematologic palliative care, research in palliative care, enrollment in palliative care, oncology, hemato oncology

## Abstract

**Background:**

Research in PC (Palliative Care) is frequently challenging for patient’s frailty, study design, professional misconceptions, and so on. Little is known about specificity in PC research on Hematologic cancer patients, who have distinct characteristics that might influence the enrollment process.

**Aims:**

What works, how and for whom, in increasing enrollment in studies in PC on patients with hematologic malignancies?

**Methods:**

Realist review: a qualitative review whose goal is to identify and explain the interaction between Contexts, Mechanisms, and Outcomes (CMOs). The theory was informed by a narrative, theory-based literature research, including an initialsystematic research, and the addition of papers suggested by experts of the field. We also used 7 interviews with experts in PC about patients with hematologic malignancies research and our own experience from a PC pilot study on patients with hematologic malignancies to refine the initial theory.

**Results:**

In our initial theory we hypothesize that:

- Access to palliative care could be beneficial to hematologic patients, even in early stages

- Hematologists tend to under-use palliative care services in general, due to unpredictable disease trajectories and cultural barriers.

- These factors may negatively impact the patients’ enrollment in PC research

We included secondary literature as narrative reviews, if they presented interesting propositions useful for our theoretical construction. 23 papers met our inclusion criteria.

We also searched for relevant CMOs impacting referral in palliative care, and we selected a list of CMOs that could be relevant also in hematology. We accordingly theorized a group of interventions that could increase the enrollment in PC research and presented them using “social exchange theory” (SET) as a theoretical framework.

Prominent researchers in PC in hematologic malignancies were interviewed on their opinion on our results, and additional CMOs.

**Conclusions:**

Before conducting research in PC on patients with hematologic malignancies, it’s probably advisable to assess:

- The perception of the different actors (physicians, nurses, other professionals involved), in particular the hematologists, in terms of pros and cons of referral to PC and enrollment in PC trials

- The existing relationship between PC and the Hematology department

Accordingly, it’s possible to tailor different interventions on the various actors and choose a model of trial to increase the perception of benefits from PC and, consequently, enrollment.

## Background

In this section we are presenting the known difficulties met when recruiting PC (palliative care) patients in research projects, and the goal of this paper: investigating how these difficulties apply to PC patients with hematologic malignances. We used the realist (see **Box 1**) approach for this, as we developed an Initial theory (presented at the beginning of the “results” section) and we “refined it” through an evidence informed process, consisting of different steps (see “data collection and analysis” in “materials and methods” section) and produced a more refined, final theory of what works, for whom and in what circumstances in enrollment of palliative care patients with hematological malignances (reported at the end of the “results” section).

**Box 1 Glossary of terms of realist methodology.**

Realism: theory-driven research approach, which produces evidence-informed theories, to better understand how an intervention works, for whom and under what circumstances, through the search for underpinning mechaninsms (“retroduction”).

CMO configuration:

Context: environmental backdrop elements of an intervention or program (ig: laws, cultural norms). Context in realist theory describes “in what circumstances and why interventions or programs ‘work’”.

Mechanism: resources offered in a specific context (ig: information) and reactions of people involved (ig: trust or engagement). It should provide an “an explanatory account of how and why programs gives rise to outcomes”.

Outcome: effects of specific mechanisms in a defined context, both intended or unintended (ig: adherence to a treatment).

Initial Rough theory (IRT): hypothesis of underpinning mechanisms in a program or intervention, usually, in the form of “if…then” statements, that need to be furtherly tested.

Refined theory: theorization resulting from the testing of IRT through the analysis of the gathered evidence.

Patients with advanced hematological malignancies suffer from a very high symptom burden and psychological, spiritual, social, and physical symptoms comparable with patients with metastatic non-hematological malignancy (1–4).

In agreement with the new World Health Organization recommendation (5) the evidence from studies performed in patients with solid tumors and hematologic patients’ symptom burden suggests that an earlier and integrated provision of specialized palliative care has the potential to improve their quality of life and reduce resource consumption through effective management of psychological and physical symptoms, appropriate relationships, effective communication, and support in decision-making. Palliative Care study design must take into account intrinsic methodological challenges, such as the unpredictability of disease progression, recruiting difficulties, and high attrition rates (6). Moreover, outcome measures that assessed the acceptance of the study by the participants were frequently absent (7) and RCT (Randomized Controlled Trial) design may be more frequently connected with people who are unwilling to be enrolled, aseven the use of words like “randomization” and “placebo” (6), can be negatively perceived by the patients. In the other hand,a language perceived as clear, and non-technical in that specific culture, and the use of words more oriented to symptom management then to palliation could have a positive impact.

Trials encountered enrollment challenges; for example, the consent approach rate in the ENABLE III trial of early versus delayed initiation of concurrent palliative care was 44%, with a variety of reasons given by approached patients for declining participation (7, 8).

Thespecialist’s opinion about the experimental arm involved in the trial proposal can also influence the enrollment (6, 9).

If they have the perception of “failing the patient”, or adding burden, or if they lack faith in the proposed intervention, when referring to palliative care, because they lack faith in the specific research or intervention proposed, fears to speak about prognosis, or perceive the enrollment procedure as too demanding for the usual care staff, this might have a negative impact on the overall enrollment (10). In their study, White et al. state that over three quarters of interviewed patients stated that they would be interested in trial participation if their doctor made it clear that he/she was keen for participation (6). The absence of symptoms can decrease patients’ motivation, and in general patients need to see some relevant potential personal gain, as the access to additional care or a better symptom management (when they are already present), or feel that their contribution can be helpful to others. Organizational factors can also have an effect, such as if the patient must attend multiple visits or travel further to receive the offered service.

Little is known about specific research in PC regarding *hematologic* cancer patients.

Studies showed heterogeneity in the population, PC intervention, disease trajectory and treatment phase (11). Only in the last 2 years some evidences on effectiveness arose on high symptomatic hospitalized patients by EL-Jawahri et al. (12).

Following the WHO recommendation, we initially developed a PC intervention integrated with standard hematological care (13). This pilot study was primarily focused on assessing the feasibility of the PC intervention. Secondary aims included exploring its acceptability by patients, professionals and caregivers and collecting preliminary information on its effectiveness. Our study design was discussed with hematology colleagues to better understand how to propose it and the inclusion criteria suitable for the feasibility trial including patients at their last active treatment (see **Table 1**).

**Table 1:** Our pilot intervention.

Our intervention: difficulties met, and initiatives taken in response
Before we started writing the protocol: • we met with the 2 referring hematologists expert in myeloma multiple and chief of department to discuss inclusion criteria of the trial. • a focus group was conducted to explore difficulties in enrollment As a result, some initiatives were placed from the start, as: • hand-delivery of written reminders for the office desk of hematologists • weekly in-person reminder, at scheduled ward’s meeting • periodical reminders to formal leaders of the ongoing trial
During the enrolment stage, Hematologists listed some difficulties: • it’s hard to keep in mind the possibility of enrollment in non-pharmacological protocol through ordinary care • the suggested timeline (*before* starting the last active treatment) for the enrollment can be an obstacle, as: - some patients potentially eligible for the intervention needed urgent access to palliation, and so were excluded from the protocol (as they couldn’t be randomized and enrolled in the study) - sometimes clinicians needed to start the allegedly last line of therapy in a really short time, making the enrollment process impossible • trial’s design was aimed to maximize safety and benefit for the patient: when control group patients asked to receive palliative care, they were immediately redirected to it. This might have negative influenced the clinicians’ perceptions of the relevance of the trial intervention.
as additional possible improvement strategies we tried to: • engage “trial champions”, as we asked to hematologists that showed particular interest in the trial to sponsor the trial enrollment • involve the formal ward’s leadership, to explore their perceptions on ongoing difficulties

However, the enrollment for this protocol has been difficult; it started in November 2018 with patients and caregivers; we enrolled 15 patients in 3 years.

It’s essential for our research team to understand the reason for this low accrual, related to patients, professionals, trial itself or organization. We believe it should be interesting to compare our experience with other realities all over the world.

In this paper we described a realist synthesis (14, 15) (read **Box 1** for details on realist methodology), based on our previous Review, a rapid review on Hematologic cancer patient and research in Palliative Care (final check March 2022) and experts ‘opinion on PC trials for hematologic cancer patients.

Eventually, We (11) integrated these data with our experience.

Hence, the aims of the current study were:

- to provide an overview of difficulties in patients enrollment in palliative care studies, specifically in hematologic malignancies, exploring the experts’ point of view, literature overview, our experience.
- to elaborate a realist synthesis of enrollment in palliative care intervention for hematologic cancer patients

The results of this study might be relevant for developing structured intervention proposals regarding hematologic cancer patients in PC trials or to give some suggestions to our colleagues involved in research protocol in this complex topic.

With this in mind, as expected by the realist approach, we aimed at producing a theoretical contribution, starting from an “Initial Rough Theory” (IRT) at the beginning of the process and finishing with a more refined version of it, as a result of our research work.

## Materials and methods

The process that we followed could be considered a process of realist synthesis; we decided to include secondary studies in our revision, which is not typical, and we tested our Initial rough theory with an independent study.

This part of the process is compatible with the realist logic, but it’s not a fixed stage of usual research strategies in realist synthesis. We considered as our guide for this manuscript the “Quality standards for realist synthesis for researchers and peer-reviewers” (16, 17) of the Rameses project.

According to realist analysis methodology, our first literature consultation aimed at the development of a rough theory (IRT), that further research and expert consultation aimed to refine the IRT, focusing on what seems to work better, for whom, and how, describing it through Context-Mechanism-Outcome (CMO) configurations (see **Box 1** “glossary of terms of realist methodology”).

The initial rough theory was based on a previous systematic revision of literature from our team (11) and our knowledge from our personal experience in conducting a trial on PC with hematologic patients (see **Table 1** “*Our intervention: difficulties met, and initiatives taken in response”*).

We then better specified our focus and decided to extend our search of possible mechanisms that might have an impact on the enrollment process to contiguous fields. In addition to the search for CMOs regarding the enrollment of hematologic patients into PC studies, we searched for articles describing CMOs relevant in the referral to palliative care in hematological patients. (Research strategy reported in **Table 2**, where we reported both the shift of focus of our research and the correspondent article selection process, as suggested in “quality standards for realist synthesis”, standard 5 and 6) (17). This is an example of “progressive focusing”, a well-established technique in qualitative research in which the focus of the inquiry is iteratively clarified by reflection on emerging data (50).

**Table 2:** Articles’ selection.

rationale of articles’ selection and correspondent shift of research’s focus	research strategy
initial literature systematic review (Tanzi, S., et al. (2020). “Early palliative care in hematological patients: a systematic literature review.” BMJ supportive & palliative care 10(4): 395-403.): aim of the review was to synthesize the evidence on the impact of early palliative care on hematologic cancer patients’ quality of life and resource use	Embase,Cochrane, CINHAL and Scopus searched for: • (early OR integrated OR simultaneous care OR concurrent) AND palliative care OR early palliative care OR simultaneous care AND (haematologic* OR haematologic* OR onco-haematologic*); english, up to 7/2/2020. - 296 articles retrieved, - 8 articles included in the review (18–25):
Second literature research for theory refinement March 2021: after developing an RCT experience our research team focused on how and why hematologic enrollment in palliative care research proved to be so difficult in our and other professionals’ experience.	Pubmed: • research[Title/Abstract] AND palliative[Title/Abstract] AND (hematologic*[Title/Abstract] OR haematologic*[Title/Abstract]) Filters: Adult: 19+ years, from 2011 – 2021: - 53 records retrieved, - 12 records included in the review (7, 12, 18, 26–34):
After interviewing 7 main experts in the fields that resulted eligible as authors of the main works retrieved in the previous articles retrieval, as appropriate in realist synthesis, we then decided to “seek out data from situations outside the program under study where it can be reasonably inferred that the same mechanisms(s) might be in operation” (Rameses project’s standards), and retrieved additional records from bibliographies, considered articles and interviewees indications, exploring the contiguous fields difficulties in hematologic research in general, and difficulties in palliative care research in general, searching for relevant CMOs for our research question(“why enrollment in palliative care studies on patients with hematologic malignancies is so difficult?”).	16 articles selected for the realist synthesis (6, 8, 9, 35–46)
After iteratively analyzing the selected articles, we decided to focus on the more specific aspects of difficulties in hematologic referral and difficulties in palliative care research on patients with hematologic malignancies, as the information and CMOs configurations retrieved in the articles about the difficulties in PC research in general where mainly already reported in the other two groups.	selected as highly contributors to our research’s question: FINAL ARTICLES’ SELECTION: 22 articles (7, 12, 18, 20, 26–33, 35–37, 42, 43, 46–49):

We derived an interview guide (see appendix 1 “the interview guide”) to collect data about the different research teams that are conducting similar studies. The interview was developed following the recommendations by the RAMESES project for “realist interviews” (17, 51).

### Data collection and analysis

**Figure 1:**
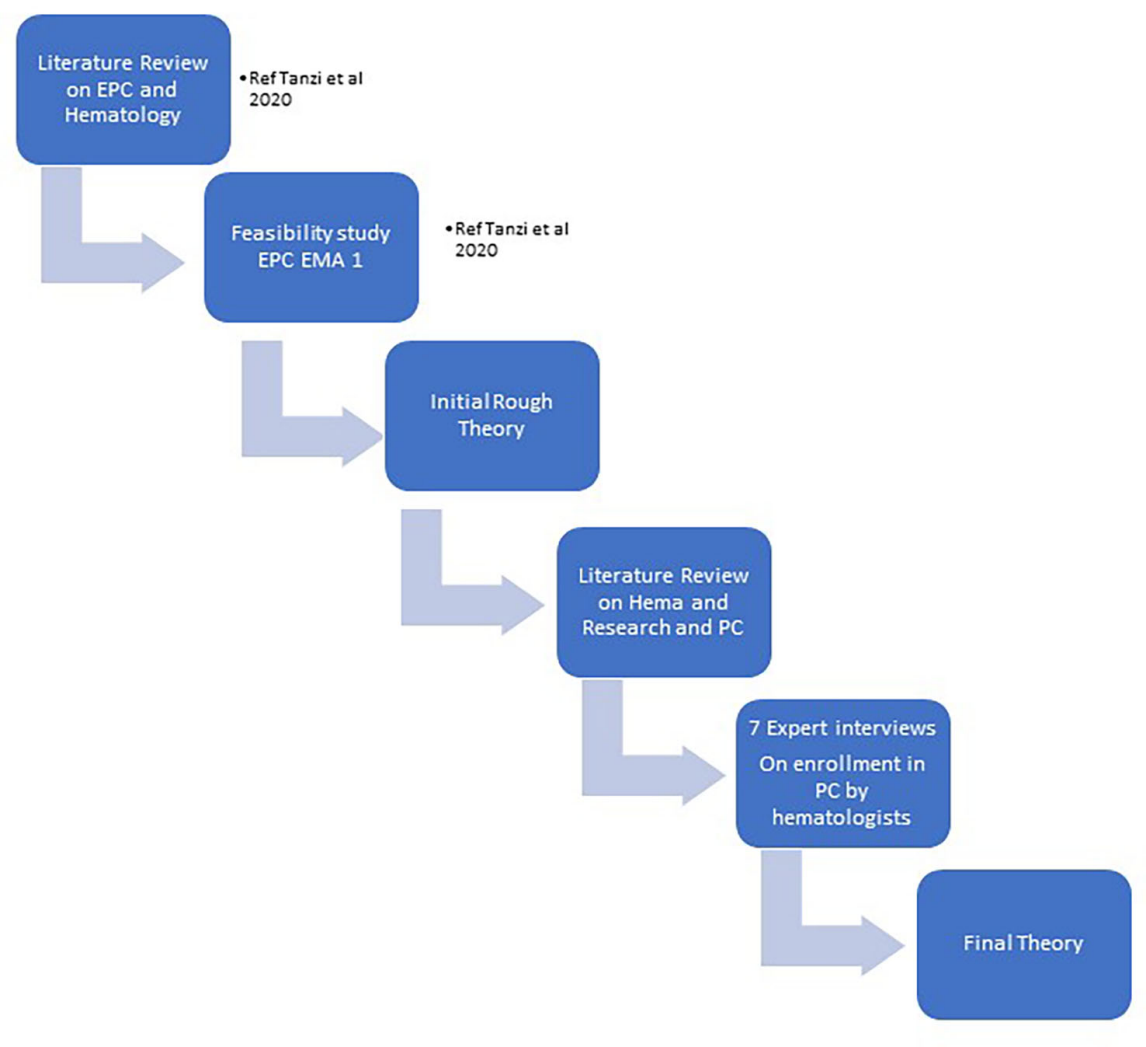
The phases of research.

Steps in developing our final theory were shown in **Figure 1** “phases of research”.

They were:

- STEP 1: we developed our IRT starting from literature review on Early Palliative Care and Hematologic cancer patients and our experience in a pilot feasibility trial
- STEP 2: we searched for relevant palliative care studies conducted with hematologic patients and for ongoing trials.

We analyzed the available materials (published papers, protocols and abstract), using an appraisal process in which we made a first selection based on abstract’s pertinence, and then a second appraisal rating the full-text articles based on their relevance (“high”, “medium”, “low”, “none”). Study characteristics (e.g. sample type and size, type of research, grade of evidence) and theoretical contribution (e.g. ‘how’, ‘why, ‘in what circumstances’) were tabulated on an Excel spreadsheet.

- STEP 3: we developed a list of the retrieved CMOs, linking them to the different studies, to have an operative summary of the main mechanism that seemed likely to have an impact on hematologic studies’ enrollments (see **appendix 2**).
- STEP 4: we developed an interview guide based on the CMOs’ list and the suggested guidelines for authors’ interviews in realist evaluations; we then contacted the authors of the research that we analyzed to gather additional information on their studies and to compare our findings with the experts’ opinion (see **appendix 1**).

In October 2020, we sent a first email to ask the availability for an interview; in December 2020 -March 2021 we conducted 7 interviews to the researchers involved in palliative care on patients with hematologic malignancies interventions. GM conducted audio-recorded phone interviews with key informants of researcher teams, purposively selected according to the following characteristics: having conducted a palliative care study on hematologic cancer patients published in literature, trials ongoing (referring to trial.gov registration, last research July 2020) or published research protocols. Two experts were also contacted based on their works presented in congresses’ abstract. The semi-structured interviews were transcribed verbatim by GM. The authors of the 2 trials ongoing did not answer to our invitation.

Both authors searched the transcripts and the articles for possible context, mechanisms, ad outcomes configurations that could emerge and refine the initial rough theory (see **Table 3**).

**Table 3:** CMOs from the interviews.

Mechanisms	*Verbatim*	Cod	Effects on enrollment +/-	already retrieved in literature research? Y/N
Early access to PC for ALL transplant patients	*.we decided that it would be a good idea to try to see if we can have all of our patients going to transplant the at least evaluated by palliative care.* *I mean, having the patient having met you and having some sort of therapeutic relationship with you, even if in the beginning when you’re seeing them, you’re focusing on and patients with myeloma on neuropathy and you’re focusing on not able to sleep. And then if things change, you’re focusing on other things, you’re what you’re talking about, doesn’t it evolves and that’s that feels natural to a patient, which I think is good.*	1	+	Y
Use other term than Palliative care	*or we called it supportive care here, not palliative care.* *We change the name for you know, we didn’t choose and we don’t use palliative care physicians, we use supportive care.* *i miei colleghi ematologi spesso presentano il servizio di cure domiciliari parlando di cure domiciliari, non di cure palliative.*	Cod 1, cod 2 4	+	y
Proposal Pc as a extra layer support	*. The goal is to figure out how you’re going to get through this better.* *You know, an extra layer of support. And we are delighted if these patients are cured.*	Cod 2, 3,6	+	y
Systematically propose PC	*Part of that also is seeing supportive care and the cancer center. They see a dietician. They see a social worker. They see a financial counselor. They see supportive care.*	1	+	y
To propose PC for its impact on outcomes	*So the idea is that it could also impact on the outcomes of the, you know, the process to not just be on the comfort of the patient*	1	+	y
Dedicated PC physician	*And here she works solely in our cancer center, basically. And so she doesn’t have to go to see heart failure patients or ICU patients. She can focus on cancer care*	1	+	y
Favorable organization	*Our cancer center and our hospital are right beside each other, so it’s literally twenty five yards away to get to the inpatient side* *And one other thing is linking the visits with other visits, like getting the palliative care visit on the same day as the oncologist visits.* *una unità di cure palliative all’interno dell’ematologia dove il paziente viene intercettato all’interno dell’ospedale*	1 4	+	y
Pc as symptoms control in first instance	*I think that that’s not going to work as well because the patients will never want to do anything more than they have to do.*	1	+	y
Good relationship between PC and hematology teams	*I have a really good relationship with the hematology oncology team, so I’m able to talk to them on a daily basis. I can just walk into their work room and say, Hey, this patient has this issue or this patient is doing really well. I’m really excited about it. So there’s a really good working relationship.*	1	+	y
Inclusion criteria included term as “incurable”		2	–	N
Hematologist do not recognize PC needs	*they told me that patients were not in a palliative state for that kind of disease.*		+	y
Developing a research protocol togheter	*And we thought that developing research together might be a great opportunity to develop collaboration and improvement in that with hematological malignancies patients. So we use research in order to improve clinical collaboration*	Cod 2, cod 3	+	y
Identify specific hematology population	*. I think it might highlight the need for them to come up with some specific patients.*	2	+	y
Starting from hematologists needs	*I think it wasn’t for symptoms management, it was more like a bed management problem,… even if it’s a hematological patients and we can manage and improve the symptoms management as well.* *Blood transfusion* *Antibiotic treatments* *Hemorrhagic events* *These are often elderly patients, with many issues, both social and... physical, and therefore they cannot access the service, they cannot come to the day hospital, and so our colleagues make requests for us... but not because they know what simultaneous palliative care is.* *But also through subcutaneous or intravenous routes, medications can be administered, medications for the disease, and we do that, so it's easier, so to speak, to entrust, how should I put it, entrust it to the group.*	2 4	+	Y
Misconception about PC by hematologist	*And when you try to say, like, you can be in palliative care situation, and still have oncological treatments. This is not something that many of them actually, they don’t really integrate … OK, it’s great for patients when we don’t have any treatment to propose.* *So, I believe it's really a communication issue among peers, meaning that, in the end, a hematologist recognizes someone who is a hematologist. But who also has skills in palliative care... and so even I, I am convinced that we...*	2 4	–	y
Having always a therapeutic line to propose	*And in hematological field, there are improvements. I mean, major improvements may be more than sort of to us. I don’t know if that can be. And so I think maybe innovations for oncological treatments might be something which is not helpful for us. Because there always. It’s always … moving that line*,	2	–	Y
Don’t talk about the prognosis	*the official reason is about the prognosis. They don’t talk about the prognosis of the patients.*	2	–	y
Local Reality/specific local context	*They [oncologists] had participated to our two earlier trials [ … ] Because of that, they were so positive about the idea of early palliative care that the idea that we were going to do a delayed trial was not very positively received [ … ]*	Cod 2, 4	–	
Caregiver opinion	*obviously in research, yeah, the the caregivers opinions are very important. And they should be maybe one point to that might improve enrollment as well.*	6	-/+	y
Using an embedded model	*So in the outpatient clinic, we were embedded in the clinic. And so from a practical standpoint … We would sort of either sequentially see the patient while they were there or sometimes we would go together.*	Cod 3	+	Y
Having similar department (pain clinic) can influence/having drug trial	*we have something kind of difficult for palliative care. I mean, like we have a pain team and palliative care team.* *, they just aren’t going to do it, because they’re so busy worrying about treating the leukemia, or maybe trying to get the patient onto a drug trial*,	Cod 2 5	–	y
Having strict criteria to defined advanced hematological cancer	*if you looked at the additional materials, you would have seen that we had very specific criteria To describe Advanced, … right and so … yeah. Yeah. And so the hematologic ones there were … chromosomal markers … There were all sort of things.* *Cioè ci sono tanti elementi che uno dovrebbe prendere per poi costruire una sorta di semaforo giallo rosso per dire questo è un paziente da segnalare…* *High risk patient*	3 4 5	- + +	y
Symptomatic patients	*this was a great, great intervention and palliative care is great, but I really didn’t need it right then; I needed it later. And so you’ll some of the patients told us they preferred not having it. Maybe it was too early for that…. They weren’t feeling symptoms. They weren’t all the stuff that we were working with them on. You know, as far as decision making and problem solving and all that stuff, those weren’t their big issues, right? That they didn’t, they didn’t have them, but they weren’t till later.* So *for these patient it was actually in my mind an easier sell, because they were already overwhelmed. They were already distressed. Some of them are already symptomatic, and so they, they appreciated any extra layer of support they can have*	Cod 3 Cod 4 Cod 6	+	y
Strength collaboration within a research	*which is what we’re teaching them in, you know, working with them in palliative care about. And these are skills and education that’s going to be helpful to them, whether they are cured or not cured. So there’s nothing harmful about what we’re doing. So they had to learn* *we had to create that culture first, before going into and doing sort of a study that focus on end-of-life for optimizing end-of-life care*	Cod 2 Cod 3 6	+	y
Previous good collaboration with hema team	*And so before I was starting this trial, we had kind of grown up together and I helped support their, their ability to do bone marrow transplant, clinically trained, all the nurses, all the problematic staff.*	Cod 3 4	+	y
Being an insider/finding a champion in the hematology team	*But these are hematologists who have... created a path, instead of going out, they have created an essentially in-hospital palliative care unit.* *that has been a very essential to the success of this study, is the fact that those of us who are leading them, are part of the leukemia and Transplant teams.*	Cod 4 5	+ +	y
Simultaneus care model	*The fear of a break, of an interruption in the relationship with the institution responsible for the patient, and therefore the "tearing" of care towards an unknown team;*	4	+	y
Sharing *crossroad* visits	*And so we have these meetings, where we call back the hematologist who was in charge, who certainly has more authority in saying, "Look, things have changed."*	4	+	y
Systematically approach all eligible patient	*The research staff were screening from the inpatient roster.*	6 5		y
Not involved the hematology in the proposal	*think that obviously impacts all of these of my studies is how do you present the fact that* *you may be randomized to usual care, and not to have these clinicians involved, and so …* *is to not rely on the oncologist for referrals.*	6 5	+	
Coaching to a standard research proposal	*a huge part of actually the challenge was training research coordinators across institutions to approach the process of describing the study, describing what palliative care is in a consistent fashion* *to have prepared a sort of a script and to train the research coordinators or any research staff about how to talk to the patient, About palliative care.*	6 5	+	y
Stress to participate for altruistic reason	*and honestly most patients sign up for my studies for altruistic reasons*	6	+	y
Not been PC an extra cost	*f these studies that the cost of healthcare is part of their inpatient Hospital stay, and so they were not receiving extra personal cost of them*,	6	+	y
Not been perceived as a survey	*he concern about being in usual care, the concerns about “I don’t wanna fill out surveys”*	6	+	y
Avoiding use jargon for randomization		5	+	y
Training in giving difficult communication for research staff	*We have actually had in-person training sessions for the research staff. So you practice that in in a pretend way as part of the training for becoming a coordinator on this on these trials.*	5	+	y

### Ethics

This Research project did not include the collection, processing, or analysis of personal or sensitive data of an interested party. Accordingly, the research did not require review or approval by the Ethics Committee. Nevertheless, specific participant protection procedures were adopted: researchers asked participants to agree to participate in the survey and interviews on a voluntary basis by email, and to give their informed consent orally during the audio registered phone call.

## Results

### Initial rough theory

We developed our IRT through a published systematic review (11) and the testing in our context through a trial (13). We tried to apply some suggested improvements during the enrolment of our research study: some attentions were planned just from the beginning of the study and others were added during the enrolment process (see **Table 1** “our intervention”).

Enrollment in palliative interventions have its difficulties, but hematology has some specific obstacles, leading to additional difficulties to enrollment and subsequent development of new high-quality knowledge.

Additional features that might negatively impact enrollment *in* PC interventions on patients with hematologic malignancies probably are:

- Difficulty in prognostication by hematologists:
- Disease development: uncertainty in its trajectory (also for the advent of potential lifesaving therapies-as CAR T-cell) and consequently on referring to PC.
- On the other end, patients suitable of a PC intervention were identified between very “end of life” population (life expectancy of days/few weeks)
- Defining target population: Difficulty to understand which hematologic population could benefit most from PC service, based on patients’ needs as perceived by hematologists
- Organizational challenges: especially for ambulatory outpatients, it’s hard to keep in mind the possibility of enrollment in non-pharmacological protocol through ordinary care. Moreover, sometimes clinicians needed to start the allegedly last line of therapy in a really short time, and palliative care evaluation and randomization was not possible

### Theory refinement process

We refined our initial theory through a) literature research for relevant mechanisms and b) interviews to experts in the fields.

We are presenting our results based both on their source of retrieval (“CMOs literature research” and “CMOs in interviews”), and as our “refined theory”, a possible global theorization of how the different CMOs might be theoretically related.

#### CMO in literature research

In our literature research, we selected some relevant mechanisms that might have an impact on the enrollment process. We hypothesize that if hematologists do not refer to PC at the same time, they don’t enroll in a palliative care trial.

So, for the aim of this project we wrote 2 tables (see **appendix 2**):

CMO on patterns on referral to PC by hematologists
CMO on specific patterns on PC research for hematologic cancer patients

##### Palliative care referral for hematologic cancer patients

This group of CMOs focuses on the difficulties of referring to PC by hematologists and the mechanisms which have an impact on it.

Some of these M regard the *model of integration* between hematology and PC and other *organizational difficulties*: strict criteria to access to hospice, for example, lack of space and time to discuss about PC, hospital culture focused on curing, being in different department and not having access 24/24 hours to PC service, could reduce referral to PC. A linear (from beginning to end) model more than a sequential one (PC only when hematologic care is concluded) could improve PC referral as having clear leadership on patients between the 2 staffs. Poor communication between staffs is detrimental even for PC referral.

*Relation between hematologist and pc professionals* with reciprocal acknowledgment could improve PC referral, not seeing referring to PC as a failure or a deskilling. Perceived self-efficacy by hematologists and misconceptions about palliative care could reduce referral to PC service. The term PC itself could be avoided. *Patient’s conditions* as asymptomatic patients or patients with unrealistic expectations could reduce the integration between the 2 staffs. Hematologic patients could have specific needs not addressed by PC and unexpected disease trajectory makes difficult to recognize PC needs. *Hematologists difficulties* to propose a consultant inside a long-time relationship with patient, late end of life discussions and unrealistic expectations from active treatments could reduce PC referral by hematologists.

##### Palliative care research for hematologic cancer patients

In this group we analyzed mechanisms suggested from the scarce literature on enrolment in PC for hematologic cancer patients (7, 18, 35–37). The mechanisms underlying the low enrollment seem to be quite similar to the well-known mechanisms in PC in general (8, 9, 38–42, 52, 53), with some more specificity regarding this subgroup as the difficulty to define a clear prognosis. Identifying patients with highest supportive needs may improve feasibility and acceptability of future primary palliative care in hematologic malignancy trials. Moreover, lack of patient interest in the topic of palliative care research also potentially affected the feasibility.

#### CMOs in interviews

The interviews with expert partially agreed with the results from the literature, but they also contributed to add some significant insight into our research question (see **Table 3** “interviews’ mechanisms” and **Table 4** “interviewers characteristics”). Experts’ interviews suggested that the initial identified population should be rich in symptoms burden to start building a collaboration with hematologists.

**Table 4:** Interviewee characteristics.

Code	Study type	setting	In/outpatient	Personal experience
Cod 1	Retrospective review	Hospital	In/out	+
Cod 2	Pilot study	Hospital	In	–
Cod 3	RCT	Hospital	In/out	+
Cod 4	Observational	Home care/ambulatory	out	+
Cod 5	RCT	Hospital	In/out	+
Cod 6	RCT	Hospital	In/out	+
Cod 7	Pilot study	Hospital	In/out	–

Consequently, in a second time, end-of-life patients could be co-managed between the two staffs, with a simultaneous approach. Moreover, being part of the hematologic team or being perceived like an insider seem to be the winning element in the RCTs realized until now.

Finally, trials with inpatients -as transplanted patients, for example - could be easier to conduct, due to the high symptoms burden and the access facility to the ward.

On the other hand, failure experience collected from the interviewed experts are described as linked to the population target definition as “incurable”, a criterion hard to recognize for hematologists.

Moreover, the hematologist point of view on Palliative Care is essential for both refer to PC and propose a PC trial.

### Refined theory

**Figure 2:**
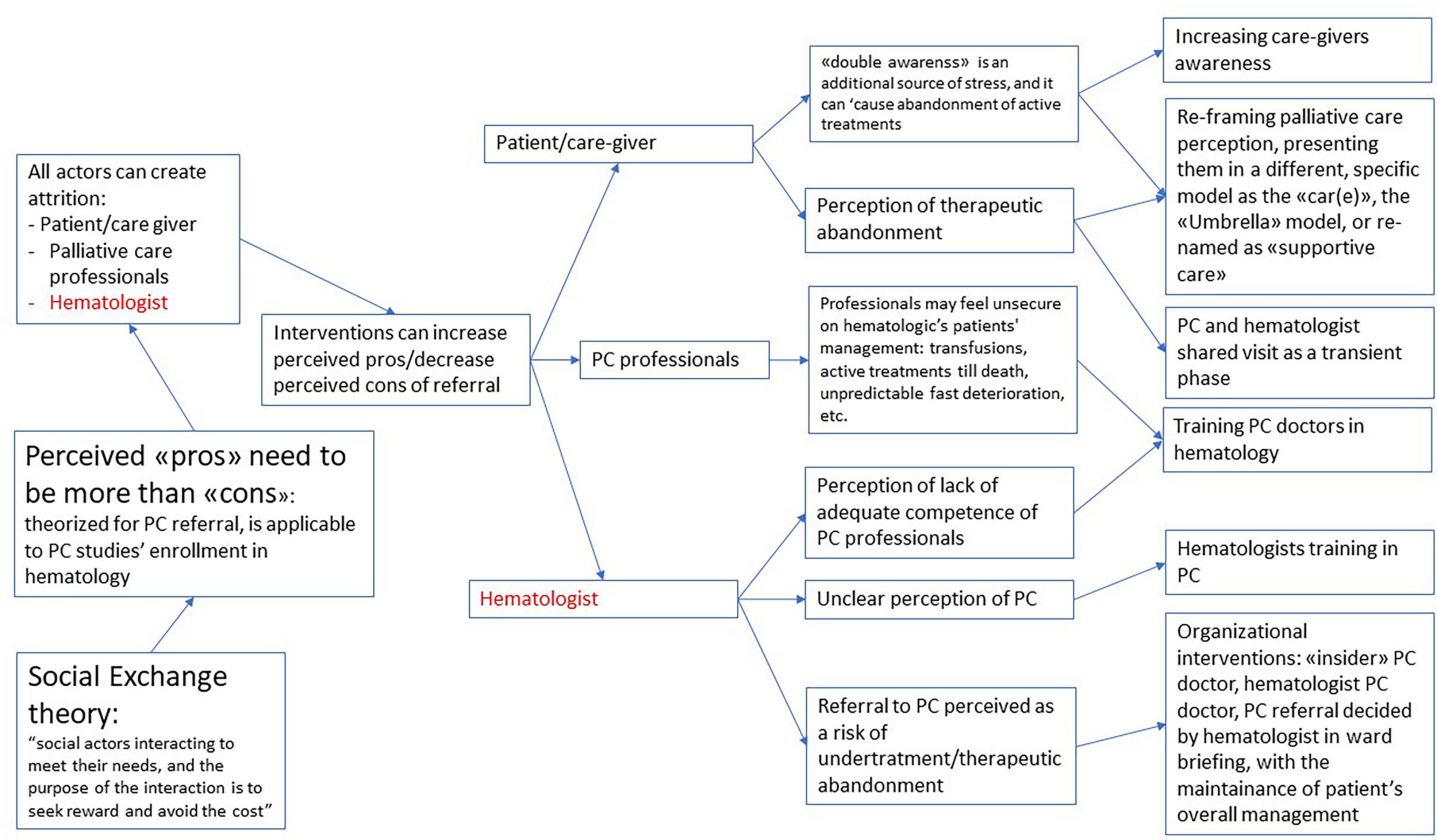
Refined theory: what works, for whom and in which circumstaces, when enrolling hematologic cancer patients in palliative care?

An important finding of this review was that ‘success features’ did not seem to be intrinsic to any specific single study design or type of research, but the result of many different interactions between different contexts and mechanisms. “Social exchange theory” by Homans was used by Salins to explain the possible problems in referral in palliative care (47), including hematology. We selected this theory as flexible and useful enough to be used to explain the problems in enrollment in PC studies in hematologic patients too. According to this interpretation, referral is a social interaction, and depends on the perception of social actors of this interaction as capable of providing a sort of reward and avoid a cost. As represented in **Figure 2**, it’s plausible that every actor involved can create attrition in the enrollment process. But as stated both in the reviewed literature and in the experts ‘opinions, it’s possible to design a study or a clinical environment to create a perception of a more favorable reward/costs relation for all the actors involved: this might be seen as the “intermediate mechanism”, on which different kind of interventions might have an impact.

It’s possible to intervene on the perception of patients and caregivers, where the “double awareness” (26) of potentially fatal development of the disease and at the same time potentially life-prolonging intervention creates a high stress. For instance, reframing their perception of palliative care through the use of a different term (as “supportive care”) (27) or the explanation of a different framework for palliative care for patients with hematologic malignancies as the “CAR(E)” or “Umbrella” model (43), or even with an explicit decision to create a higher involvement of the care giver in partial substitution of the patient.

It’s also possible to increase the self-efficacy of palliative care doctors, through specific hematologic training, considering the specific differences of this patients’ population.

But it’s highly likely that the more relevant actor in the process might be the hematologist. Many possible interventions might lead to a better perception of the advantages of PC referral.

An unclear perception of referral as a possible source of undertreatment might be addressed with organizational adjustments, as having a PC hematologist, or a palliative care consultation that is discussed in the ward meeting and keeps the patient under the hematologic management.

**Figure 3:**
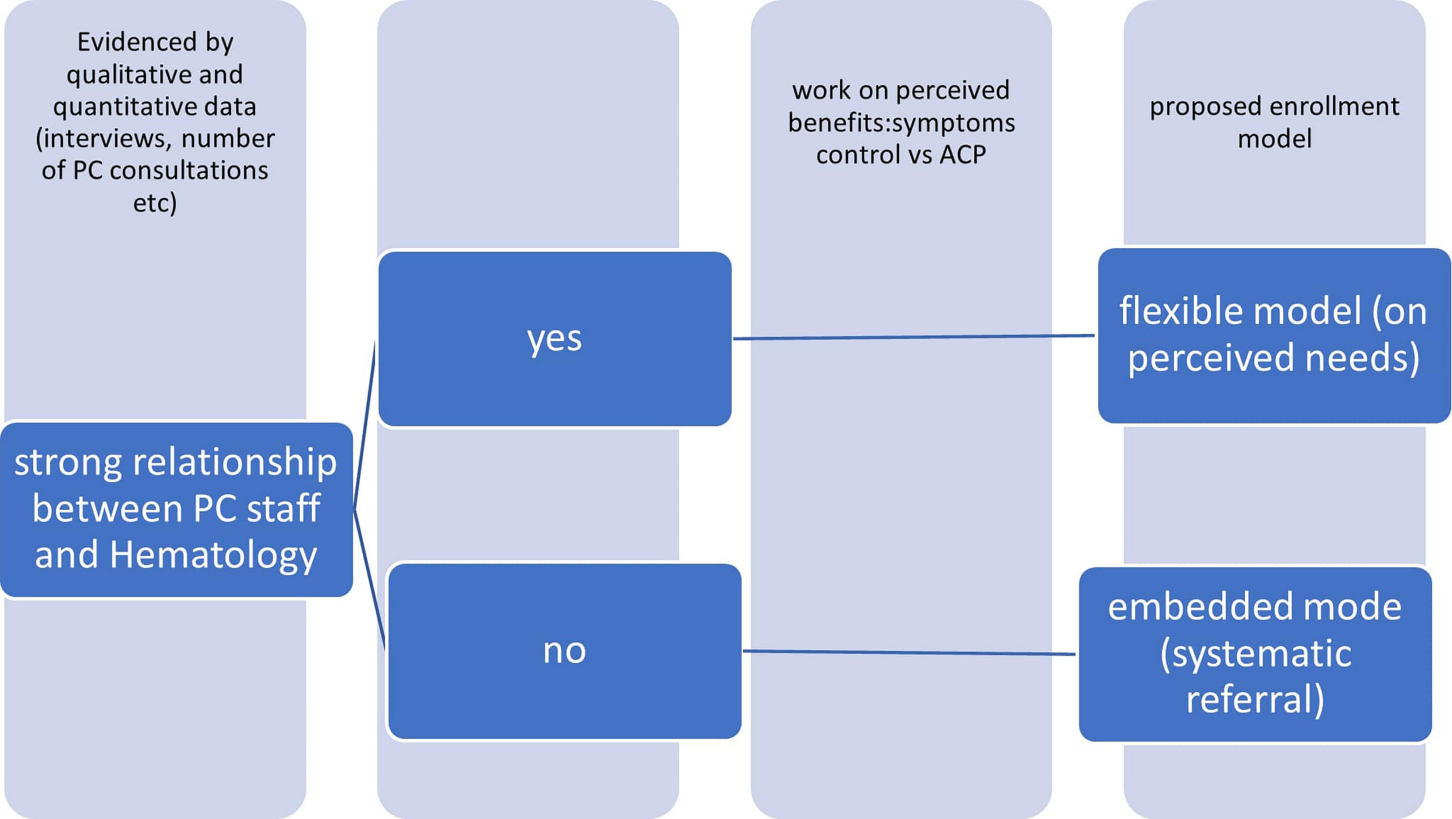
Teams' relationship and enrollment.

As a consequence, (see **Figure 3**) the perception of the different actors might be the key element to lead to an intervention modulated on the characteristics of the specific environment in which the study might be developed, in particular the perception of hematologists. A stronger, already existing relationship between the two teams might imply the chance of working on highly complex needs. On the other end, a new relationship might require an easier task to start, as addressing highly symptomatic patients (ig, patients undergoing transplantation).

## Discussion

This synthesis from literature and experts ‘opinions allows us to deepen the topic of enrolment in PC trial in hematologic cancer patients.

As highlighted by our results, the problem of enrolling hematological patients in palliative care trials overlaps with dynamics inherent in the referral to PC services by hematologists, in general.

We defined our general refined theory as a “ecological theory of enrollment in palliative care research on patients with hematologic malignancies”.

As a refinement to our initial list of CMOs impacting the enrollment process, we selected the “social exchange theory” (SET) of Salins (47) as a relevant model for our theoretical construction. In his SET, he theorizes that oncologists need to have a clear perception of the advantages that they might get from the referral to palliative care, and that these advantages need to outbalance the costs.

This model is useful to explain the difficulty of enrollment in palliative care intervention in hematologic patients too and could be integrated with other theoretical aspects specific for this field. We face in hematologic patients the specific difficulty of “double awareness” (as theorized by Gerlach (26)) that puts the patients and the caregivers on a specific tension due to the double possibility of having a rapid deterioration of health conditions to death or getting to a disease-free period of time thanks to the medicines. Applying the SET model to hematology intervention, we might see how this aspect of “double awareness” needs to be managed both by health professionals and patients and caregivers. Health professionals will then be assessing their pros and cons of referral, knowing that the costs of the referral might result in less awareness of curing possibilities and less focus on available treatments.

Another relevant CMO that we added to our initial theory, is that palliative care needs the PC professionals to be really flexible, to increase referral to PC of patients with hematologic malignancies, searching for the most suitable model for their environment. While we listed several aspects that could have an impact and need to be addressed while designing the intervention, if we start from the SET theory, it seems safe to theorize that every intervention should start from the assessment of the perception of the hematologists of the possible advantages and disadvantages of the referral to palliative care. A first distinction should be between interventions that are built on a strong relationship between PC staff and hematologists, and interventions that are developed independently from an already relationship between the teams. Often, these interventions might implicitly be designed to build a better relationship by the leaders of the program.

Quantitative elements could be informative on the level of integration; while qualitative data could help selecting the elements that could be addressed by an intervention aimed at reaching a more cooperative environment.

The successful experiences reported of enrollment of hematologic patients in palliative care were all based on a previous positive experience of cooperation between the two teams (7, 18). It might be unlikely that the enrollment process could be successful in a context where the intervention itself aims at obtaining a better interaction between the two teams.

Some interventions are possible and seem more likely to work, and all of them might be interpreted as an effort to increase the pros/cons ratio and the perception of the palliative care contribution in the hematologists.

Mere technical improvements (such as a remembering email or a phone call from the researcher) as well as simply hypothesizing a different study design (42) seem to not be able to solve the question and might lead to miss the more relevant points.

The contamination of knowledge with a Palliative care/hematology model that is not only integrated but embedded (44) would respond both to organizational problems and to those related to misconceptions on PC; both expert interviews and data from literature confirm this suggestion.

The health care professionals gate keeping-where the professionals don’t recognize PC needs- was recognized as a barrier to PC enrolment by the literature (42) and seems to be logically applicable in the hematologic setting too. An integrative model “fluctuant, flexible and based on patients’ needs”, where these needs are detected by hematologists has been suggested as a possible model of optimal integration (3). But it might be beneficial to consider the possibility of an even more embedded model, where PC is almost “forced” in hematology ward’s daily work. It could minimize the burden of the intervention both for patients and clinical staff and overcome the difficulties by hematologist to recognize PC needs especially in asymptomatic patients. Moreover, having a PC physician/nurse as a member of the hematologic team could lead to perceive palliative care as a routine component of the patient care.

According to this, an additional mechanism that might be beneficial in terms of integration is the training of hematological professionals in palliative care and in understanding deeply the palliative care approach, while training palliative care practitioners as well to the specificities of the hematological patient, as suggested by many authors (26, 28–30, 45).

Our experts’ interviews also suggested that enrolling only symptomatic patients could be a more initial intervention; however, an early approach also for asymptomatic patients could change the culture/improve the acceptance between palliative care professionals and hematologist. The referral not only for physical needs but also social, psychological, ethical and spiritual ones, should be learnt and improved (26, 46).

Unpredictable course of hematologic malignancies could negatively impact the enrollment.

Using objective and systematic criteria for enrollment (as conducing a first assessment on the list of transplants, or having an automatic flagging and reporting of patients with bad prognosis criteria) would avoid this lack. Artificial intelligence has had a growing improvement for this kind of problems (54).

## Limitations

The overall quality of a review is strongly influenced by the quality of the primary studies considered. The difficulty in gathering firsthand data on palliative care patients is the very reason why this approach might be interesting, as we tried to produce a theoretical contribution based on what is known, what is guessable and what is not known to help navigate this difficult field.

A realist review is an evidence-informed review, who is only partially evidence based, as part of the effort in this specific type of review is trying to produce a theoretical contribution from the available data. We attempted to suggest possible solutions and useful links between what is perceived as connected in this field, trying to start from making explicit what is “obvious” for the researchers in the field but not so obvious for the readers.

This approach limits the exact generalizability of our suggestions, but encourages researchers to try and confirm or challenge our hypothesis, as expected by realist methodologies.

## Conclusions

The referral to PC- as the enrollment in a PC trial - should be tailored on patients’ needs and recognizing these palliative care needs is not simple for Hematologists.

To recognize the relationship between PC staff and Hematology is mandatory to propose the right approach, an integration flexible model or on an embedded model.

Consequently, we suggest that expected outcomes should be different, based on a preliminary evaluation of the context of the intervention: while an intervention based on a new relationship might have as a starting stage the aim to address complex symptoms control, and might also explicitly be part of a wider intervention that might result in building stronger relationships between the different stakeholders. On the other side, when a strong, previous relationship between the staffs is already present, it might increase the chance to address more complex topics as advance care planning.

## Data availability statement

The original contributions presented in the study are included in the article/**Supplementary Material**. Further inquiries can be directed to the corresponding author.

## Ethics statement

Ethical review and approval was not required for the study on human participants in accordance with the local legislation and institutional requirements.

## Author contributions

Both authors contributed to all parts of the manuscripts. In particular, ST worked more on background and discussion and GM worked more on “methods” and results. All authors contributed to the article and approved the submitted version.
