# Supplementary material for: Doing palliative care research on hematologic cancer patients: A realist synthesis of literature and experts’ opinion on what works, for whom and in what circumstances
Source: Front Oncol. 2023 Mar 27;13:991791. doi: 10.3389/fonc.2023.991791 (PMC10083487; doi:10.3389/fonc.2023.991791)
Supplement: Supplementary file 1 [file DataSheet_1.docx]

# Enrollment process in general

1. What is/have been **your role** in this study?

we had the chance to see the published material of your study; since we’re trying to better understand the underlying mechanisms of how the enrollment in palliative care RCTs works in hematology, I’d like to know more on your study about the enrollment process.

How would you describe your **enrollment process**?

(if needed: check question after the answer: For what we understood from your [study protocol/published papers] and what you told us, your study is an RCTs, and [ig, the hematologist] presents the study to the patient during usual care; then [ig, an independent researcher] explains the details and collects consent.)

1. What have been the **results** of the enrollment process, till now?

what did you **expect** it to be? is there any sensible difference? ig: you recruited far less than expected;

1. The evidence suggests that enrollment in palliative care RCTs might meet some additional difficulties compared to other fields. What is your opinion about it? did you meet **any particular difficulty** in the enrollment process?
2. In your opinion, what makes the enrollment process work **better**?
3. Has it been any unexpected outcome or **unexpected factor** in the enrollment process? we’re interested in both positives or negatives.
4. Did you notice any particular difference in the success of the enrollment process on **different targets**? ig, different patients responded in different ways in the enrollment process.

##

1. Is there any other **similar or derived protocol** that you are planning to do in the present or future?

Is there any qualitative study conducted ongoing or conducted on this RCT study?

Our idea is that the enrollment process in palliative care hematologic RCTs might work in different ways with different patients or in different settings.

We found some useful points in the current literature on RCTs in palliative cares, even if there is a gap of evidence on hematology.

**I’ll cite some aspects that might have an impact on the enrollment process, and I’d ask you to tell me if, and how, you find it relatable to your specific study.**

# Focus on specific topics

## Trial design

Some elements related to **trial design** seem to have an impact.

1. How is the trial design presented and how do you think this might impact your enrollment process?

| Did the enrollment explanation include the words “randomisation”, “placebo control”, “blinding”? |  |
| --- | --- |
| Do you think that randomization somehow impaired the enrollment process on the patient side? o on the side of the clinician that presented it? |  |
| (is it presented as a drug trial or pain study?) |  |
| is the chance of possible additional financial strain presented? |  |
| Is the trial introduced by the familiar staff or by an independent researcher? |  |
| Do you have a dedicated resource (i.e. a researcher that takes care for the study)? |  |

## retaining autonomy

The topic of **retaining autonomy** might have an impact on the decision to participate to a trial.

1. How, if at all, is the topic of patient autonomy addressed during the presentation if the study?

| (if applicable in this study) did you stress the chance that the study might help the patient in retaining autonomy? |  |
| --- | --- |

## significant other’s opinion

For what we know, the opinion of others seems to be relevant in the patient decision.

1. How, if at all, do you address the **caregiver’s opinion**?

| did you actively explore the caregiver opinion on the study as a usual practice? |  |
| --- | --- |
| did you address possible caregiver resistances, if applicable? |  |
| did you reassure the caregiver that, if needed, they can interrupt their participation at any time, especially if the patient’s condition might make the participation more difficult for him/her? |  |

## other doctors opinion

The **opinion of the other doctors** seems to have an impact on the enrollment process.

1. How, if at all, do you address the other doctors opinion? What impact does it have on your enrollment?

| Did you routinely explore the opinion of the doctor that is managing the usual care of the patient about the trial? |  |
| --- | --- |
| DO you think that the patient perceives his/her participation to the trial as something that is appreciated from his/her usual care team? |  |
| Did you identify and involve in the enrollment process the clinical leaders? How? |  |
| Did you identify and involve any “champions” (i.e. doctors that are more interested to the study and act as a positive example for the others)? How? |  |

## Personal gain

The patient perception of potential for personal gain seems to have an impact.

1. What, if any, possible **potential for personal gain** is presented to your patients in the enrollment process?

could you describe how you presented this gain? Do you think it had any impact?

| is a possible relief from present symptoms presented as a possible gain? |  |
| --- | --- |
| is the chance of having closer contact with the current staff (physician, nurse…) presented as a possible gain? |  |
| Is the chance of receiving additional specialistic care be presented as a possible gain? |  |

## Altruism

The patient perception of **contributing to an altruistic action** might have a positive impact on the enrollment process.

1. is this aspect presented to your patients, and in that case, how?

| is the chance of helping other patients with your own contribution presented in the enrollment process? |  |
| --- | --- |
| does the RCT staff usually address this topic, expressing gratitude for the contribution that the study could give to the other patients and families? (possible retain factor) |  |

## Age

1. Does patients **age** have any impact on your enrollment process?

Did you perceive any difference in the enrollment related to the patient age?

## Communication

1. In introducing the trial, did you use a language specifically studied to be easily understood by the patient?
2. Has the study been advertised to the patient through newsletters or media?

## additional material

Sometimes in these studies the interviewer that is enrolling the patients have **a written manual to the interview**.

1. Is this your case? would it be possible to have it as material for our study?

Is there **any additional material** that could help us understand how your recruitment process works? i.g. letters you sent to your hematologists, additional instructions for them or for enrollers…

## closing

1. Do you think there is **anything else** we should know in order to better understand how your enrollment process works?

Thank you so much for your time and your valuable contribution.
